# The Domestic Crime

**Published:** 1870-02

**Authors:** 


### THE DOMESTIC CRIME.

Of all the sins which could be committed by living man, there were only three for which there was no atonement; only three for which there was no sacrifice appointed in the theocratic government of the Jews. And it is a very remarkable fact that, to this day, the Israelites, in all lands, are most wonderfully exempt from these crimes. In fact, they stand out in honorable prominence in these regards among all nations, tribes, and kindreds of the earth, showing their noble devotion to religious sentiment, which may well be followed, as a model, by all who profess the Christian name. These three crimes tend to subvert the very foundations of society. They overturn all law, human and divine, and defy all consequences in this world and in the world to come, making men abandoned and reckless, returning them to anarchy and a barbarism which is an utter disgrace to humanity.

IDOLATRY

was one of these crimes; a crime against the Israelitish nation, and a crime against God: hence, for it there was no atonement, and its punishment was death. No man who has ever read the Bible, Catholic or Christian, need have pointed out to him how idolatry subverts the very foundations of civilized society.

MURDER

was another of the unpardonable crimes, and it was announced, "He that sheddeth man's blood, by man shall his blood be shed." There was only one thing in mitigation of this horrid sin, and that was with a view to secure justice in every case—to prevent premature punishment. If a man killed another, he was allowed a temporary suspension of this punishment by having the privilege of fleeing to a city of refuge, several of which were established as such in different parts of the nation. Hence, all that a man-slayer had to do was to run faster than any one else, and enter the gates of the city before the avenger of blood could overtake him. But even this exemption was allowed only in case the killing was accidental, or not deliberately done, only for him "which killeth any person at unawares;" but, to determine this, whether it was "at unawares" or not, the man-slayer had to "stand before the congregation in judgment."

### ADULTERY.

The third crime for which there was no pardon, and for which the derelict "shall surely be put to death," was in fidelity in the marriage relation, and in the New Testament it is plainly stated that thieves and idolaters and adulterers "shall not inherit the kingdom of God."

### DIVORCE.

In the Christian dispensation a divorce could not be procured except for proven adultery, and whoever married a "divorced person" was guilty of adultery.

While there was no provision of atonement or pardon in the "Jewish Dispensation," no relief from the punishment of death, swift and inevitable, there is, in the Christian system, a broader line, a more merciful arrangement, in the assurance that, superior to all sacrifice of bird or beast, was that of the Son of Man, in that "the blood of Jesus Christ cleanseth from ALL sin." At the same time, it was plainly stated, on all suitable occasions, that the thief, the murderer, the idolater, and the adulterer were in the same category—were upon the same platform, which stands over an eternal perdition.

The sanctity of the marriage relation, as interpreted in the Bible, in its fullest sense, is at the foundation of a true civilization, and the more rigidly it is adhered to the more elevated, the more refined, the purer, the happier will society be.

There is one sweeping and speedy method, and practicable too, of meeting and vanquishing the rapidly growing evil of a loose interpretation of the marriage vow. Let each man and each woman regard the violation of another's hearth and home with the same alarm, with the same uncompromising hostility, with the same horror, as if their own home were thus invaded. This will enable them to set their faces, as with a flint, against all apologists for it. Let them be ready, on all occasions, in public, in private, and in secret, too, each man and each woman, one to another and always, put their foot down promptly and determinedly upon the very slightest apology for the great, yes, one of the greatest crimes against man, his country, and his God. Let ladies everywhere, and of all ages and conditions, refuse association, and even recognition, of those, whether male or female, and let parents close their portals against all who dare venture, in the most gingerly terms, an apology for looseness of interpretation in this direction.

It is a very remarkable thing that, within a very few years, three alarming evils have been growing, as if in partnership, especially in our large cities—Sabbath profanation, a less appreciation of the Bible as a Divine communication, and the facility of divorce. This very morning's paper announces that eighty divorces were granted at the session of a single court in New Hampshire. "No prayer" was lately appended to a marriage notice in Hillsborough; and if New England, with her grand histories and accomplishments in all that is elevating to man and society and the nation, thus leads off in this direction, it is very sure that alarm ought to be promptly taken by all good men, and means be adopted toward staying the tide of domestic calamity, and of national deterioration; for, in proportion as marriage is held in low consideration, in such proportion will society become corrupt, depraved, bestial; and with the moral corruption bodily disease makes equal pace, and intemperance and premature death complete the horrible history.

